# A Clinical Journey Mobile Health App for Perioperative Patients: Cross-sectional Study

**DOI:** 10.2196/20694

**Published:** 2021-02-08

**Authors:** Stijn J Willems, Michel W Coppieters, Yvette Pronk, Miranda J F Diks, Klaas W A P van der Heijden, Servan Rooker, Gwendolyne G M Scholten-Peeters

**Affiliations:** 1 Department of Human Movement Sciences Faculty of Behavioural and Movement Sciences Vrije Universiteit Amsterdam Amsterdam Netherlands; 2 Menzies Health Institute Queensland Griffith University Brisbane & Gold Coast Australia; 3 Department of Orthopaedics and Research Kliniek ViaSana Mill Netherlands; 4 Department of Epidemiology and Social Medicine University of Antwerp Antwerp Belgium

**Keywords:** eHealth, mHealth, applications, musculoskeletal, user-friendliness, rehabilitation, usability, patient education, technology, disability, feasibility, adherence

## Abstract

**Background:**

Mobile eHealth apps are important tools in personal health care management. The Patient Journey app was developed to inform patients with musculoskeletal disorders during their perioperative period. The app contains timely information, video exercises, and functional tasks. Although the Patient Journey app and other health apps are widely used, little research is available on how patients appreciate these apps.

**Objective:**

The primary aim of this study was to evaluate the user-friendliness of the Patient Journey app in terms of its usability and the attitudes of users toward the app. The secondary aim was to evaluate positive and negative user experiences.

**Methods:**

A web-based questionnaire was sent to 2114 patients scheduled for surgery for a musculoskeletal disorder. Primary outcomes were usability (measured with the System Usability Scale) and user attitudes regarding the Patient Journey app (assessed with the second part of the eHealth Impact Questionnaire). The secondary outcomes were evaluated with multiple choice questions and open-ended questions, which were analyzed via inductive thematic content analyses.

**Results:**

Of the 940 patients who responded, 526 used the Patient Journey app. The usability of the app was high (System Usability Scale: median 85.0, IQR 72.5-92.5), and users had a positive attitude toward the Information and Presentation provided via the app (eHealth Impact Questionnaire: median 78.0, IQR 68.8-84.4). The app did not adequately improve the users’ confidence in discussing health with others (eHealth Impact Questionnaire: median 63.9, IQR 50.0-75.0) or motivation to manage health (eHealth Impact Questionnaire: median 61.1, IQR 55.6-72.2). Three core themes emerged regarding positive and negative user experiences: (1) content and information, (2) expectations and experiences, and (3) technical performance. Users experienced timely information and instructions positively and found that the app prepared and guided them optimally through the perioperative period. Negative user experiences were overly optimistic information, scarcely presented information about pain (medication), lack of reference data, insufficient information regarding clinical course deviations and complications, and lack of interaction with clinicians.

**Conclusions:**

The Patient Journey app is a usable, informative, and presentable tool to inform patients with musculoskeletal disorders during their perioperative period. The qualitative analyses identified aspects that can further improve the user experiences of the app.

## Introduction

eHealth and mobile health (mHealth) tools have the potential to enhance the quality of health care and to reduce health care costs [1]. Consequently, the use of eHealth and mHealth can play an important role in supporting personal health management by encouraging healthy behavior and improving adherence and self-management [2,3]. mHealth can have additional value because only a limited amount of medical information can correctly be remembered after a consultation, and mHealth apps can be used at any time and any place [4-6]. This can enhance information recall and adherence to health instructions [5,7,8]. Furthermore, recent research shows that education provided to patients through their smartphone may improve their levels of knowledge, medication or treatment adherence, satisfaction, and clinical outcomes, as well as having a positive effect on health care economics [9].

Previous research showed that the use of mHealth apps is well appreciated by users during the perioperative period in different health care settings [10,11]. Reported advantages are the patient’s sense of being looked after, enhancement of patient-centered care, cost-effectiveness, and the increased efficiency of health care services [10,11]. However, to date, the user experiences of health care apps for the perioperative guidance of musculoskeletal surgeries have not yet been evaluated.

Based on these advantages, we evaluated the user experience of a widely used mHealth app called the Patient Journey app for patients with musculoskeletal disorders. The app provides timely information, exercises tailored to the condition and recovery, and functional tasks. The app was developed with the assumption that it addresses the patients’ needs better at specific time points and improves self-management compared to traditionally provided information.

Even though the app is widely used by over 100 hospitals and clinics in more than 20 countries, evidence about how patients appreciate this app is not yet available. Before an effectiveness study can be performed, the user-friendliness of the app needs to be assessed. Therefore, the primary aim of this study was to evaluate the user-friendliness in terms of usability and the attitudes of users toward the app. The secondary aim was to explore positive and negative user experiences.

## Methods

### Study Design

This was a cross-sectional user-friendliness study using digital surveys. The study was approved by the local medical ethics committee of Vrije Universiteit Amsterdam (VCWE-2017-005). All patients provided digital informed consent prior to participating in the study.

### Recruitment

Participants were recruited in a multidisciplinary clinic (Kliniek ViaSana). Patients were eligible if they were older than 18 years and undergoing surgery for a musculoskeletal disorder. All patients were routinely informed about the app by the medical team, a brochure, and a banner in the waiting room. Patients were included if they used the Patient Journey app during their operative period and completed the web-based survey.

### The Patient Journey App

The template of the Patient Journey app was developed by Interactive Studios [12]. The content was developed specifically for the various health care paths in the clinic by the medical team and can be downloaded for free on a mobile device. The app aims to provide optimal patient information and to improve adherence and self-management.

**Figure 1:**
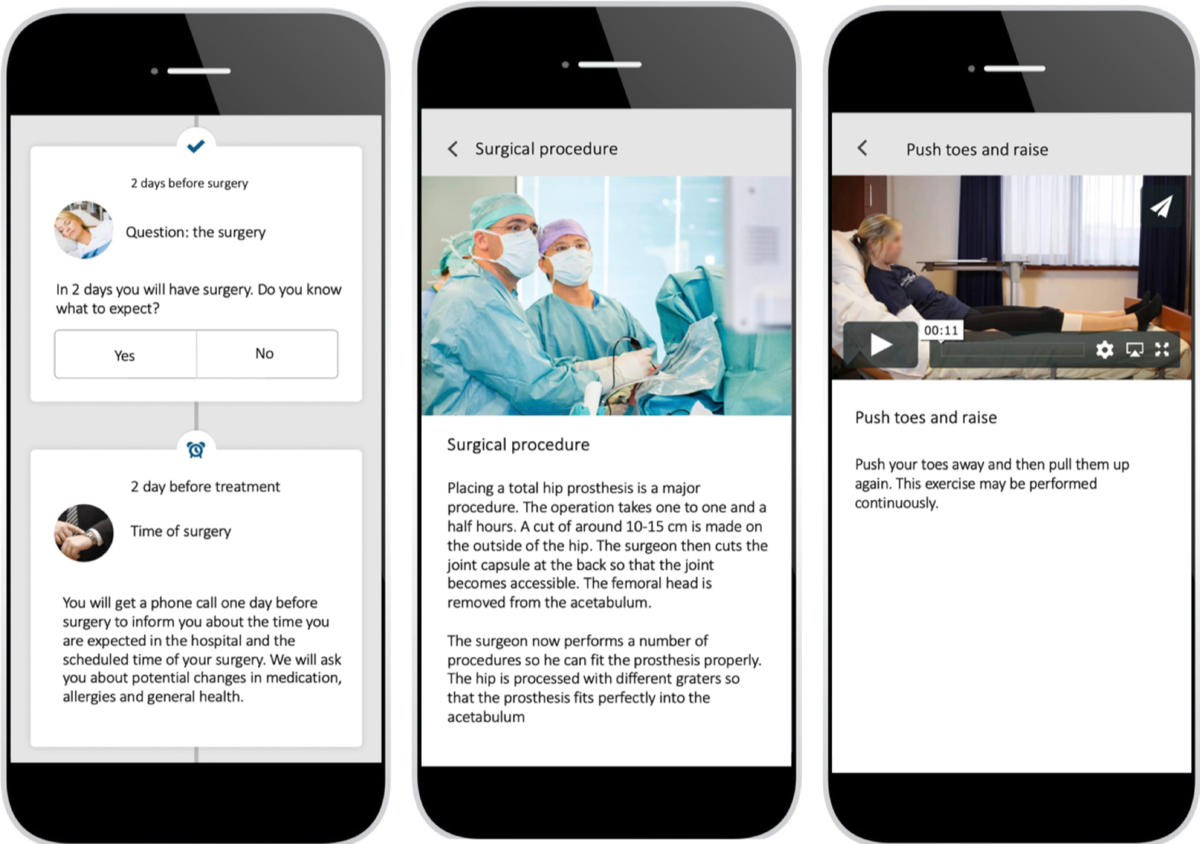
Patient Journey user interface.

The different health care paths in the app included total hip replacement, knee replacement, anterior cruciate ligament reconstruction, knee arthroscopy, high tibial osteotomy, lumbar diskectomy, rotator cuff repair, acromioplasty, femoral osteotomy, patellar stabilization, Morton neuroma, hallux valgus/rigidus, exostosis, and talocrural arthrodesis. The app is divided into 5 categories: (1) general information about the clinic and the surgeons, (2) preoperative medical and practical information (eg, medical information and anatomy, preoperative exercises, procedures), (3) information about the stay in the hospital (eg, anesthetics, surgical intervention, exercises, advice to be active), (4) homecoming information (eg, information about possible complications, medication, sleep), and (5) information about the rehabilitation process (eg, exercises, functional instructions). App users can decide to receive push notifications. All health care paths contained specific videos with exercises and functional instructions. An example of the user app interface is presented in Figure 1.

### Data Collection

Eligible participants were invited by email. The email contained a link to the digital survey. Data were collected by MailPlus (Spotler), a program designed to manage surveys [13]. Eligible participants who did not complete the survey after 1 week received an electronic reminder. Completion of the survey took approximately 15 to 20 minutes.

### Primary Outcome Measurements

The primary outcomes were (1) usability and (2) specific attitude of eHealth users toward the app. Usability was measured with the System Usability Scale (SUS) [14,15]. The SUS is a reliable and robust 10-item questionnaire and scores on a 5-point Likert scale from 1 (strongly disagree) to 5 (strongly agree) [14,16]. The total SUS score (0 to 100) can be interpreted as not acceptable (0-64), acceptable (65 to 84), or excellent (85 to 100) [17,18]. The attitude of eHealth users toward the app was measured with part 2 of the eHealth impact questionnaire (eHIQ), which includes 3 subscales: (1) Confidence and identification (9 items), (2) Information and presentation (8 items), and (3) Understanding and motivation (9 items) [19]. The eHIQ uses a 5-point Likert scale ranging from 1 (strongly disagree) to 5 (strongly agree). *Confidence and Identification* measures to what extent using the app has affected the confidence of app users in discussing and managing their health with others and whether individuals could identify with others who use the app [19]. *Information and Presentation* measures the ease of use from the user’s perspective [19]. *Understanding and Motivation* measures whether respondents felt reassured, understood their condition, and felt motivated to manage their health [19]. We transformed the total scores for each subscale to a scale of 0 to 100. A score of 65 or higher was considered as a positive attitude with higher scores representing a more positive attitude toward the app [20,21]. All subscales have good internal consistency, test-retest reliability, and construct validity (Cronbach α=.88-.90) [19,20].

### Secondary Outcome Measurements

The secondary outcomes were positive and negative user experiences. These were measured by overall satisfaction with the app, most appreciated and used parts of the app, satisfaction with the amount of information provided, whether the app was recommendable, reusability, supportiveness, and strengths and limitations of the app. *Satisfaction with the app* was evaluated with a numeric rating scale ranging from 0 (absolutely not satisfied) to 10 (absolutely satisfied). *The most appreciated and used parts of the app* and *the amount of information provided* were evaluated with multiple choice questions. Supportiveness, whether the app was recommendable, and reusability were measured with a 5-point Likert scale. *Supportiveness* was defined as the extent to which the respondent felt that the app was supportive in addition to the information given by health professionals and ranged from 1 (very poor) to 5 (excellent). Whether the app was *recommendable* was defined as ranging from 1 (not recommendable) to 5 (highly recommendable). *Reusability* was defined as the extent to which the respondent would use the app again if they had another surgery and ranged from 1 (strongly disagree) to 5 (strongly agree). Strengths and limitations were gathered via open-ended questions.

### Statistical Analysis

Descriptive analyses were performed to present patient characteristics and user-friendliness outcomes. Data were checked for normality using the Q-Q plots, histograms, and the Kolmogorov-Smirnov test. For the primary outcomes, significant differences between the different health care paths were tested by 1-way analysis of variance with Tukey posthoc tests (for continuous variables with a normal distribution) or the Kruskal-Wallis H test with Dunn posthoc tests (for continuous variables with a violation of normality). Posthoc Bonferroni correction was applied for multiple comparisons. For all statistical tests, α=.05 was used to determine statistical significance. All analyses were performed in SPSS (version 25.0; IBM Corporation). The positive and negative user experiences of the app were analyzed by descriptive statistics. Strengths and limitations were analyzed by using inductive thematic content analysis by 2 investigators (SJW, GGMSP) [22]. The thematic content analysis was inductive which means that no preexisting theory was imposed on the analysis. Two investigators reviewed the entire data set independently to get familiar with the responses. Subsequently, they coded the data independently and generated themes in a consensus meeting. In a second meeting (SJW, GGMSP, MWC), consensus was reached.

## Results

### Study Population

**Figure 2:**
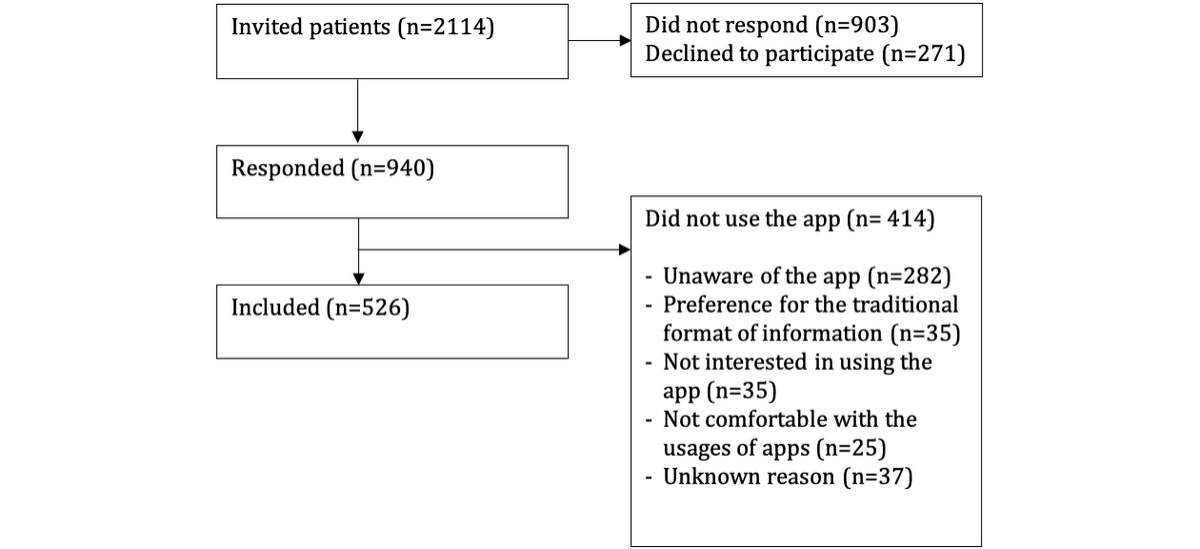
Study flowchart.

The survey was sent to 2114 possible participants, of whom 940 (46.7%) responded, 271 (13.4%) declined to participate, and 903 (39.9%) did not respond (Figure 2). Of the 940 participants who responded, 526 (56.0%) had used the app during their perioperative period.

The median age of the app users was 59.0 years (IQR 50.0-66.0), and 267 (50.8%) were female. Table 1 shows the patient characteristics of the app users and the number of participants in the different health care paths. More people who used the app were younger (*P<*.001), more educated (*P*=.01), and more frequently in paid employment (*P<*.001) compared to those who did not use the app.

**Table 1:** Patient characteristics of app users.

Variable		App users (n=526)
**Gender, n (%)**		
	Male	259.0 (49.2)
	Female	267.0 (50.8)
Age in years, median (IQR)		59.0 (50.0-66.0)
**Educational level, n (%)**		
	Low (lower vocational education)	124.0 (23.6)
	Middle (high school or secondary vocational education)	227.0 (43.2)
	High (higher professional education and/or university)	175.0 (33.2)
Duration of symptoms before surgery in months, median (IQR)		22.0 (7.0-36.0)
**Paid employment, n (%)**		
	Yes	320.0 (60.8)
	No	206.0 (39.2)
**Health care paths, n (%)**		
	Total hip replacement	89.0 (16.9)
	Knee replacement	164.0 (31.2)
	Anterior cruciate ligament reconstruction	56.0 (10.6)
	Knee arthroscopy	47.0 (8.9)
	High tibial osteotomy	23.0 (4.4)
	Lumbar diskectomy	17.0 (3.2)
	Rotator cuff repair	30.0 (5.7)
	Acromioplasty	14.0 (2.7)
	Rest group^a^	86.0 (16.3)

^a^Rest group includes shoulder arthroplasty, femoral osteotomy, patellar stabilization, Morton neuroma, hallux valgus/rigidus, exostosis, talocrural arthrodesis.

### Primary Outcomes

**Figure 3:**
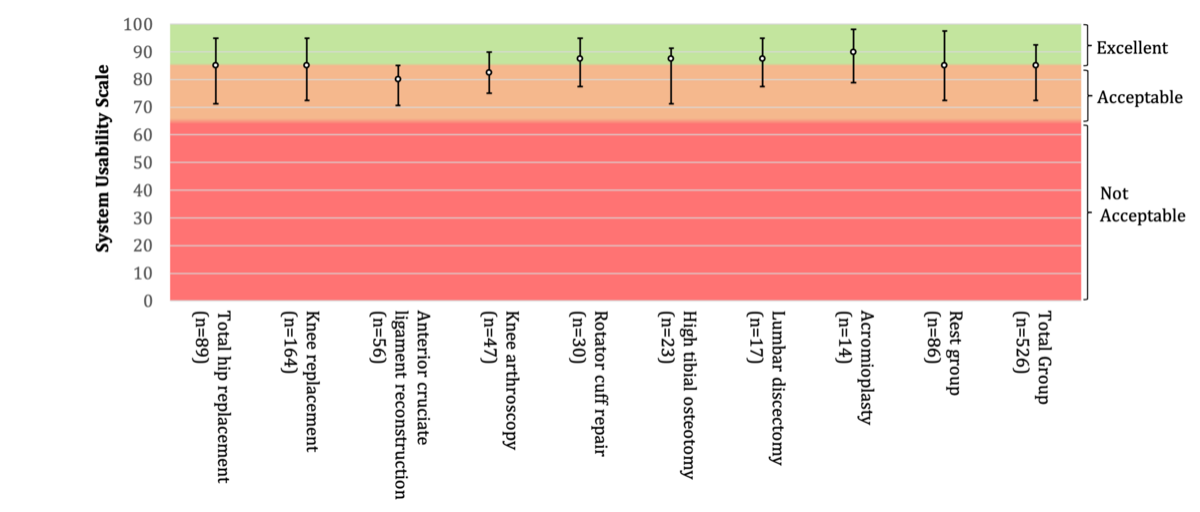
Usability.

**Figure 4:**
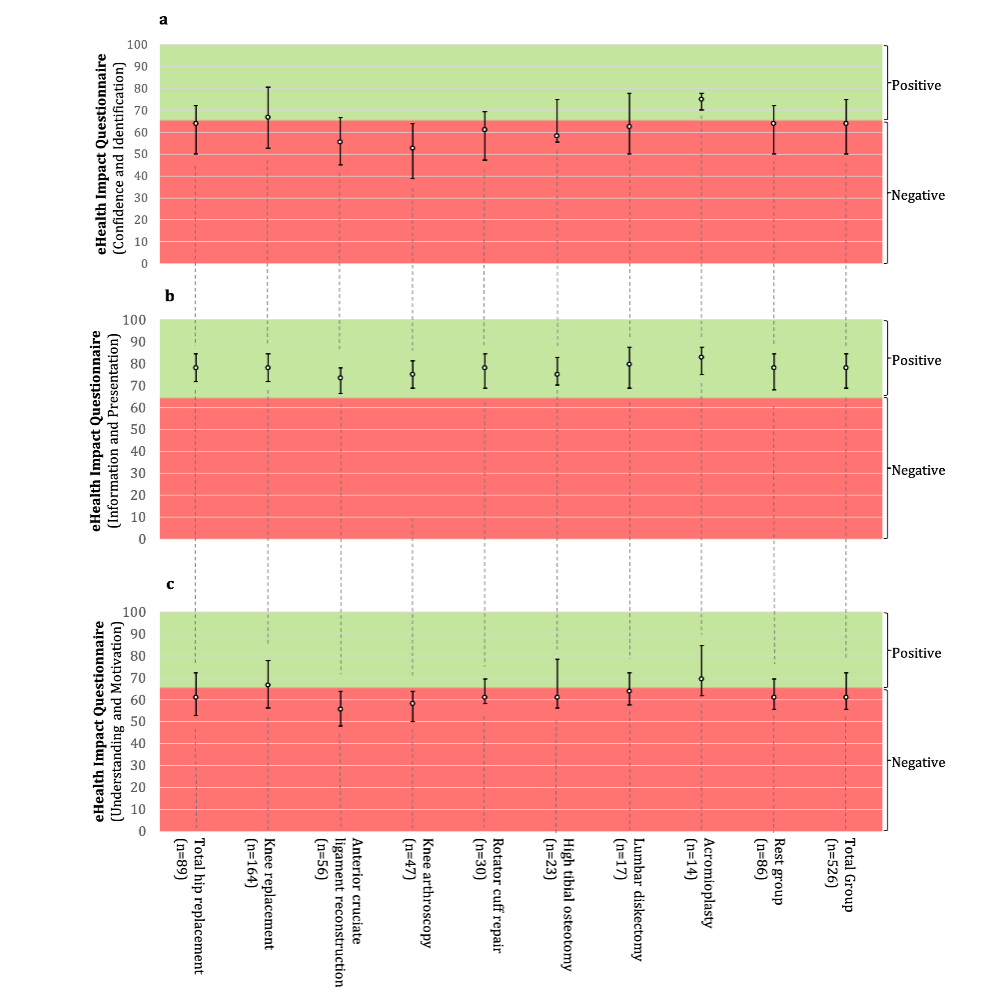
Attitude toward the app: (a) Confidence and Identification, (b) Information and Presentation, and (c) Understanding and Motivation.

Participants rated the app as highly usable (SUS: median 85.0, IQR 72.5-92.5; Table 2; Figure 3), and they had positive attitudes regarding information and presentation (eHIQ Information and Presentation: median 78.1, IQR 68.8-84.4; Table 3, Figure 4a). No significant differences between different health care paths were observed for usability (χ^2^_8_=15.5, *P*=.07).

**Table 2:** System Usability Scale scores (0 to 100).

Health care paths (n=526)	Usability, median (IQR)
Total hip replacement (n=89)	85.0 (71.3-95.0)
Knee replacement (n=164)	85.0 (72.5-95.0)
Anterior cruciate ligament reconstruction (n=56)	80.0 (70.6-85.0)
Knee arthroscopy (n=47)	82.5 (75.0-90.0)
High tibial osteotomy (n=23)	87.5 (77.5-95.0)
Lumbar diskectomy (n=17)	87.5 (71.3-91.3)
Rotator cuff repair (n=30)	87.5 (77.5-95.0)
Acromioplasty (n=14)	90.0 (78.8-98.1)
Rest group (n=86)^a^	85.0 (72.5-97.5)
Total group	85.0 (72.5-92.5)

^a^Rest group includes shoulder arthroplasty, femoral osteotomy, patellar stabilization, Morton neuroma, hallux valgus/rigidus, exostosis, talocrural arthrodesis.

**Table 3:** Attitude toward the app (eHealth Impact Questionnaire, 0 to 100).

Health care paths (n=526)	Confidence and Identification, median (IQR)	Information and Presentation, median (IQR)	Understanding and Motivation, median (IQR)
Total hip replacement (n=89)	63.9 (50.0-72.2)	78.1 (71.9-84.4)	61.1 (52.8-72.2)
Knee replacement (n=164)	66.7 (52.8-80.6)	78.1 (52.8-80.6)	66.7 (56.3-77.8)
Anterior cruciate ligament reconstruction (n=56)	55.6 (45.1-66.7)	73.4 (66.4-78.1)	55.6 (47.9-63.9)
Knee arthroscopy (n=47)	52.8 (38.9-63.9)	75.0 (68.8-81.3)	58.3 (50.0-58.3)
High tibial osteotomy (n=23)	61.1 (47.2-69.4)	78.1 (68.8-84.4)	61.1 (58.3-69.4)
Lumbar diskectomy (n=17)	58.3 (55.6-75.0)	75 (70.3-82.8)	61.1 (56.3-78.5)
Rotator cuff repair (n=30)	62.5 (50.0-77.8)	79.7 (68.8-87.5)	63.9 (57.6-72.2)
Acromioplasty (n=14)	75.0 (70.1-77.8)	82.8 (75.0-87.5)	69.4 (61.8-84.7)
Rest group (n=86)^a^	63.9 (50.0-72.2)	78.1 (68.0-84.4)	61.1 (55.6-69.4)
Total group	63.9 (50.0-75.0)	78.1 (68.8-84.4)	61.1 (55.6-72.2)

^a^Rest group includes shoulder arthroplasty, femoral osteotomy, patellar stabilization, Morton neuroma, hallux valgus/rigidus, exostosis, talocrural arthrodesis.

Participants stated that using the app did not increase their confidence in discussing and managing health with others and their feeling of identification with others (eHIQ Confidence and Identification: median 63.9, IQR 50.0-75.0) (Table 3, Figure 4a). They did not feel more reassured, did not understand their condition better, and did not feel more motivated to manage their health by using the app (eHIQ Understanding and Motivation: median 61.1, IQR 55.6-72.2; Table 3, Figure 4c). Significant differences between the various health care paths were found for the Confidence and Identification subscale (χ^2^_8_=44.6, *P<*.001), Information and Presentation (χ^2^_8_=17.3, *P*=.03), and Understanding and Motivation (χ^2^_8_=35.4, *P<*.001) subscales (Table 4). Posthoc Bonferroni comparisons showed that participants who underwent anterior cruciate ligament reconstruction scored lower than participants who underwent knee replacement (*P*<.001) or acromioplasty (*P*=.03), and similarly, participants who underwent knee arthroscopy scored lower than participants who underwent knee replacement (*P*<.001) or acromioplasty (*P*=.02) on the Confidence and Identification subscale. Participants who underwent an anterior cruciate ligament reconstruction (*P*<.001) or a knee arthroscopy (*P*=.03) scored lower than people who underwent a knee replacement on the Understanding and Motivation subscale.

**Table 4:** Comparison results of the health care paths.

Health care path comparison		*P* values		
		Confidence and identification	Information and presentation	Understanding and motivation
Difference between the health care paths		*<*.001	.03	*<*.001
**Bonferroni posthoc analysis**				
	Anterior cruciate ligament reconstruction vs knee replacement	*<*.001	—^a^	*<*.001
	Anterior cruciate ligament reconstruction vs acromioplasty	.03	—	—
	Knee arthroscopy vs knee replacement	*<*.001	—	.03
	Knee arthroscopy vs acromioplasty	.02	—	—

^a^Not tested because there was no difference between the health care paths.

### Secondary Outcomes

App users reported a median score of 9.0 (IQR 8.0-9.0) for overall satisfaction with the app. The delivery of timely information (244/526, 46.4%) and the exercise videos (135/526, 25.7%) were the most appreciated parts of the app; 93% (475/526) would recommend the app to other patients, 86.1% (453/526) found the app supportive in addition to the information given by health professionals, and 87.3% (459/526) found the amount of information exactly enough. They appreciated the information about the stay in the hospital the least and the preoperative information the most (Multimedia Appendix 1).

The results of the inductive thematic content analyses are shown in Table 5. Important strengths related to the theme *content and information* were the clear information and instructions, timely information, and clear videos with exercises and instruction. Participant 219 wrote, “I knew exactly which exercises or activities I was able to perform each day.” Limitations belonging to this theme were that information about complications and pain medication use was lacking, an abnormal clinical course was scarcely presented, and information was not completely in line with the information provided by the medical specialist and not always up to date. Participant 293 wrote: “I found the timeline too optimistic and the information given was based on a protocol that did not fit with my situation.” Participant 27 responded, “I missed information about pain medication use.” Important strengths related to the theme *expectations and experiences* were the guidance and preparation for the surgery and rehabilitation, additional supervision and the usefulness of the app. Participant 377 responded, “The app helps you what you may expect and when.” Participant 30 wrote, “The app gave me the confidence in the journey.” Experienced limitations were that the app was not entirely personalized and missed reference data from peers. Participant 52 stated, “adding comparisons with others could provide more confidence in my personal recovery.” Participant 373 wrote, “Recovery is based on the average patient and not the individual one.” Strengths regarding the theme *technical performance* were the simplicity of downloading the app and receiving of push notifications. Patient 379 wrote, “I liked the easy way in which push notifications could be switched on and off.” Limitations were that the app sometimes jumped back and did not continue with the current phase, interaction with clinicians and access to personal electronic health records. Participant 195 stated, “It would be nice to have insight in my personal health records and the possibility to ask questions via the app.”

**Table 5:** Results of the inductive thematic content analyses.

Core theme and strengths		Limitations
**Content and information**		
	Clear information and instructions Timely information Useful to read back information Clear videos with exercises and instructions	Too optimistic information Information about complications, pain medication use and an abnormal course are scarcely presented Not completely in line with the information by the medical specialist Information was not always up-to-date
**Expectations and experiences**		
	Optimal guidance/preparation for surgery and rehabilitation Additional supervision Easy to use Clear expectations and guidelines	Not entirely personalized No reference data from peers
**Technical performance**		
	Simplicity to download the app and receive a push notification	No interaction with clinicians No access to personal electronic health record App jumped back to a previous phase instead of continuing with the current phase

## Discussion

### Principal Results

We aimed to evaluate user-friendliness in terms of usability and attitudes of users toward the Patient Journey app. The secondary aim was to evaluate positive and negative user experiences. Indicated as the main findings, the usability of the Patient Journey app scores excellent and users have positive attitudes toward the Information and Presentation provided via the app. However, the app did not adequately improve confidence in discussing health with others and motivation to manage health. These outcomes differed between the various health care paths with lower scores in the anterior cruciate ligament reconstruction path and knee arthroscopy path. Most users would recommend the app to other patients and found the app supportive in addition to the information given by health professionals

The results of the thematic analyses provided insight into potential reasons why the Confidence and Identification and Understanding and Motivation subscale scores were below the recommended value [20,21]. Lack of personalized information, protocols based on the average patient, no interaction with clinicians, and missing reference data of peers were potential reported explanations. Previous research showed that the usage of interactive systems, videoconferencing sessions, and phone counselling favors in improving physical function, disability, and pain in comparison to conventional methods of information delivery following total knee and hip replacement [23]. Adding advanced telerehabilitation functions, such as including personal logs with appointments and a more personalized prognosis, or chat interactions with a physician or physiotherapist could probably increase a positive attitude of users toward the app.

Moreover, overly optimistic information, the scarcity of information about pain medication use, and how to act in case of a complication or deviation of the described clinical course could have led to the lower scores on the Confidence and Identification and Understanding and Motivation scales. Recent studies have shown that mHealth apps are promising tools in the guidance of pain control and opiate use and are effective in reducing pain medication intake [24,25]. It is therefore assumed that implementing pain measurements and content how to reduce pain medication into the app could reinforce a positive attitude of users toward the app.

An interesting finding is that participants who underwent an anterior cruciate ligament reconstruction and knee arthroscopy scored more negative on the Confidence and Identification and the Understanding and Motivation scale compared to other specific health care paths. Additional posthoc analyses revealed that participants in these groups were significantly younger than the other participants. Previous research also showed that middle-aged and older users pay more attention to their health issues and are more motivated to take action by using mHealth to avoid illness and stay healthy [26]. Therefore, we assume that younger patients are more confident in their capabilities, less motivated to manage their health, and less focused on specific health management.

Furthermore, following an intensive guided rehabilitation program after anterior cruciate ligament reconstruction could lead to higher levels of motivation and a better understanding of their condition. This may reduce the need for an app.

### Comparison With Prior Work

Although the Patient Journey app is widely used and implemented, no previous study has assessed its user-friendliness. Other research described the user-friendliness of various types of mHealth interventions having dissimilar purposes in different health care settings [27-32]. These studies [27-32] also demonstrated that mHealth apps are highly feasible and acceptable to users. No previous studies assessed the user-friendliness of mHealth tools for the perioperative period for musculoskeletal surgery. A recent systematic review [10] evaluated patients’ experiences on the use of perioperative mHealth apps; these authors found that mHealth can serve as an important tool for patient engagement in education about their condition and procedure. Moreover, mHealth apps can reduce inconsistencies between information given by health care providers [10]. Although the information provided and instructions were one of the strengths of the Patient Journey App, our qualitative analysis showed that the information provided was not always in line with that provided by the medical specialist. Comparable with our findings, reported weaknesses for perioperative mHealth use were patients’ lack of confidence, lack of personalized information, and often overly optimistic information which could lead to an overestimation of the patients’ course [10]. The timely information as provided by the Patient Journey app helps people to comprehend information and has positive effects on the patients’ levels of knowledge, satisfaction, clinical outcomes, and health care economics [9].

A general strength and important motivator for mHealth users is the accessibility of specific information that could increase knowledge about their condition [31,33]. Nevertheless, an important concern regarding trustworthiness is that this information is not always up-to-date and valid [31]. Other important factors in line with those in previous research are the lack of personalization, peer support, and integration of functionalities that enhance the interaction with clinicians [30,31]. To increase the relevance of app use, it is preferable that mHealth apps include diverse functions that enable patients to personalize and tailor them to meet their needs [31,32]. Furthermore, peer support can enhance patient socialization by providing social support, and facilitating 2-way communication with clinicians could increase patient engagement and therefore seems to be a great promise of mHealth [31,34]. In contrast to our findings, mHealth apps for patients with chronic diseases can increase feelings of managing health-related behavior by making users feel more reassured and empowered [27,31]. Most of our participants, however, did not feel more confident in managing their health by using the Patient Journey App. Potential differences could be explained by the type of participants (people with chronic diseases versus people with musculoskeletal disorders scheduled for surgery) and engagement in self-management (people who undergo musculoskeletal surgery may have less need to be engaged in self-management, especially during the stay in the hospital compared to patients with chronic health issues) [27,31]. Patients who are highly engaged in self-management experience the use of mHealth apps as more beneficial than others [31].

### Limitations

This study has several limitations. First, the number of participants in the different health care paths varied, and this could have led to imprecise results in health care paths with small sample sizes. Second, we used inductive thematic content analyses based on open-ended questions for the secondary outcomes. Semistructured interviews could have helped to deﬁne areas that could be further explored and would have given more detailed information about some themes [35]. The representativeness of the study might be biased as participants who used the app were statistically significantly younger (*P<*.001), higher educated (*P*=.01), and had more paid jobs (*P<*.001) compared to those who did not use the app. Moreover, most of our participants belonged to the middle-age group. It is unclear whether the results would have been different in younger or older age groups as different age groups may have different experiences of app usability and different expectations for how apps should function [26].

Despite these limitations, we believe that this study does provide novel insights into the user-friendliness of the mHealth app in the perioperative musculoskeletal period and that the results are of clinical importance for app users, clinicians, mHealth app developers, and researchers.

### Conclusion

The Patient Journey app is a usable, highly informative, and presentable tool to inform patients with a musculoskeletal disorder during their perioperative period. For participants in most health care paths, using the app did not improve their confidence in discussing their health or reassurance in managing their health. However, the development of utilities that can offer reference data from peers, interaction with clinicians, and more insight into pain could further increase the user-friendliness of the app.

## Appendix

Multimedia Appendix 1 Secondary outcomes.

## Abbreviations

eHIQ: eHealth Impact Questionnaire
mHealth: mobile health
SUS: System Usability Scale
