# Supplementary material for: A Clinical Journey Mobile Health App for Perioperative Patients: Cross-sectional Study
Source: JMIR Hum Factors. 2021 Feb 8;8(1):e20694. doi: 10.2196/20694 (PMC7899805; doi:10.2196/20694)
Supplement: Multimedia Appendix 1 [file humanfactors_v8i1e20694_app1.docx]

**Appendix 1.** Secondary outcomes

|  | Total hip replacement (n=89) | Knee replacement (n=164) | Anterior cruciate ligament reconstruction (n=56) | Knee arthroscopy  (n=47) | High tibial osteotomy (n=23) | Lumbar discectomy (n=17) | Rotator cuff repair  (n=30) | Acromioplasty (n=14) | Rest Group (n=86) ^a^ | Total  (n=526) |
| --- | --- | --- | --- | --- | --- | --- | --- | --- | --- | --- |
| **Overall satisfaction with the app (0-10 (median (IQR))** | 9 (8-10) | 9 (8-10) | 9 (8-9) | 9 (8-9) | 9 (8-9.5) | 9 (8-9) | 9 (8-9) | 8 (9-10) | 9 (8-9) | 9 (8-9) |
| **Most appreciated parts of the app n (%)** |  |  |  |  |  |  |  |  |  |  |
| 1. Delivery of information at the right time | 38 (42.7) | 69 (42.1) | 27 (48.2) | 24 (51.1) | 14 (60.9) | 14 (82.4) | 11 (36.7) | 5 (35.7) | 42 (48.8) | 244 (46.4) |
| 1. Sending push notifications | 2 (2.2) | 10 (6.1) | 4 (7.1) | 3 (6.4) | 1 (4.3) | 1 (5.9) | 1 (3.3) | 70.0 | 5 (5.8) | 27 (5.1) |
| 1. Exercise videos | 27 (30.3) | 45 (31.5) | 16 (28.6) | 9 (19.1) | 6 (26.1) | 0.0 | 10 (33.3) | 7 (50.0) | 13 (15.1) | 135 (25.7) |
| 1. Supported instruction videos | 13 (14.6) | 29 (17.7) | 5 (8.9) | 6 (12.8) | 2 (8.7) | 2 (11.8) | 6 (20.0) | 1 (7.1) | 16 (18.6) | 78 (14.8) |
| 1. Contact details | 2 (2.2) | 2 (1.2) | 2 (5.4) | 1 (2.1) | 0.0 | 0.0 | 0.0 | 1 (7.1) | 4 (4.7) | 13 (2.5) |
| 1. Search function | 2 (2.2) | 4 (2.4) | 1 (1.8) | 2 (4.3) | 0.0 | 0.0 | 0.0 | 0.0 | 2 (2.3) | 11 (2.1) |
| **Most usable category of the app n (%)** |  |  |  |  |  |  |  |  |  |  |
| 1. General information category | 27 (30.3) | 55 (33.5) | 8 (14.3) | 0.0 | 1 (4.3) | 1 (5.9) | 6 (20.0) | 2 (14.3) | 23 (26.7) | 123 (23.4) |
| 1. Preoperative category | 34 (38.2) | 29 (17.7) | 16 (28.6) | 28 (59.6) | 6 (26.1) | 4 (23.5) | 10 (33.3) | 2 (14.3) | 27 (31.4) | 156 (29.7) |
| 1. Hospital stay category | 3 (3.4) | 4 (2.4) | 0.0 | 3 (6.4) | 0.0 | 0.0 | 0.0 | 0.0 | 5 (5.8) | 15 (2.9) |
| 1. Home staying category | 8 (9.0) | 18 (5.9) | 19 (33.9) | 8 (17.0) | 3 (13.0) | 4 (23.5) | 3 (10.0) | 2 (28.6) | 14 (16.3) | 81 (15.4) |
| 1. Rehabilitation category | 17 (19.1) | 58 (19.1) | 13 (23.2) | 8 (17.0) | 13 (56.5) | 8 (47.1) | 11 (36.7) | 2 (42.9) | 17 (19.8) | 151 (28.7) |
| **Recommend the app to other patients n (%)** |  |  |  |  |  |  |  |  |  |  |
| 1. Totally not recommendable | 0.0 | 2 (1.2) | 0.0 | 1 (2.1) | 0.0 | 0.0 | 0.0 | 0.0 | 1 (1.2) | 4 (0.8) |
| 1. Not recommendable | 2 (2.2) | 2 (1.2) | 0.0 | 1 (2.1) | 0.0 | 0.0 | 1 (3.3) | 0.0 | 3 (3.5) | 9 (1.7) |
| 1. Nor not recommendable, nor recommendable | 7 (7.9) | 11 (6.7) | 5 (8.9) | 5 (10.6) | 1 (4.3) | 0.0 | 0.0 | 1 (7.1) | 8 (9.3) | 38 (7.2) |
| 1. Recommendable | 52 (58.4) | 89 (54.3) | 42 (75.0) | 33 (70.2) | 13 (56.5) | 13 (76.5) | 18 (60.0) | 4 (28.6) | 52 (60.5) | 316 (60.1) |
| 1. Strongly recommendable | 28 (31.5) | 60 (36.6) | 9 (16.1) | 7 (14.9) | 9 (39.1) | 4 (23.5) | 11 (36.7) | 9 (64.3) | 22 (25.6) | 159 (30.2) |
| **Amount of information n (%)** |  |  |  |  |  |  |  |  |  |  |
| 1. Too little | 6 (10.8) | 13 (7.9) | 10 (17.9) | 5 (10.6) | 1 (4.3) | 2 (11.8) | 4 (13.3) | 1 (7.1) | 11 (12.8) | 53 (10.1) |
| 1. Exactly enough | 80 (86.7) | 145 (88.4) | 45 (80.4) | 40 (85.1) | 22 (95.7) | 15 (88.2) | 26 (86.7) | 13 (92.9) | 73 (84.9) | 459 (87.3) |
| 1. Too much | 3 (2.5) | 6 (3.7) | 1 (1.8) | 2 (4.3) | 0.0 | 0.0 | 0.0 | 0.0 | 2 (2.3) | 14 (2.7) |
| **Re-use the app n (%)** |  |  |  |  |  |  |  |  |  |  |
| 1. Strongly disagree | 3 (3.4) | 11 (6.7) | 1 (1.8) | 2 (4.3) | 0.0 | 1 (5.9) | 0.0 | 0.0 | 5 (5.8) | 22 (4.2) |
| 1. Disagree | 2 (2.2) | 6 (3.7) | 0.0 | 3 (6.4) | 0.0 | 0.0 | 0.0 | 0.0 | 3 (3.5) | 15 (2.9) |
| 1. Nor disagree, nor agree | 9 (10.1)) | 11 (6.7) | 9 (16.4) | 1 (2.1) | 1 (4.3) | 0.0 | 1 (3.3) | 0.0 | 4 (4.7) | 36 (6.9) |
| 1. Agree | 18 (20.2) | 23 (14.0) | 22 (40.0) | 17 (36.2) | 6 (26.1) | 10 (35.3) | 7 (23.3) | 1 (7.7) | 16 (18.6) | 116 (22.1) |
| 1. Strongly Agree | 57 (64) | 113 (68.9) | 23 (41.8) | 24 (51.1) | 23 (69.9 | 17 (58.8) | 22 (3.3) | 12 (92.3) | 58 (67.4) | 335 (63.9) |
| **Supportive to health care n (%)** |  |  |  |  |  |  |  |  |  |  |
| 1. Very poor | 0.0 | 4 (2.4) | 0.0 | 0.0 | 0.0 | 0.0 | 0.0 | 0.0 | 0.0 | 4 (0.8) |
| 1. Poor | 0.0 | 2 (1.2) | 0.0 | 2 (4.3) | 0.0 | 0.0 | 0.0 | 0.0 | 4 (4.6) | 8 (1.5) |
| 1. Fair | 11 (12.4) | 12 (7.3) | 15 (26.8) | 6 (12.8) | 2 (8.7) | 1 (5.9) | 3 (10.0) | 1 (7.1) | 10 (11.6) | 61 (11.6) |
| 1. Good | 57 (64.0) | 102 (62.2) | 37 (66.1) | 36 (76.6) | 17 (73.9) | 13 (76.5) | 21 (70.0) | 6 (42.9) | 62 (72.1) | 351 (66.7) |
| 1. Very good | 21 (23.6) | 44 (26.8) | 4 (7.1) | 3 (6.4) | 4 (17.4) | 3 (17.6) | 6 (20.0) | 7 (50.0) | 10 (11.6) | 102 (19.4) |

N = number of patients, IQR = Inter Quartile Range, Education level^a^: Low = Lower vocational education; Middle = High school and/or secondary vocational education; Rest Group^b^ = includes shoulder arthroplasty; femoral osteotomy; patellar stabilisation; Morton’s neuroma; hallux valgus/rigidus; exostosis; talocrural arthrodesis; combined due to the low number of participants in each group.
